# Contribution of the Resting-State Functional Connectivity of the Contralesional Primary Sensorimotor Cortex to Motor Recovery after Subcortical Stroke

**DOI:** 10.1371/journal.pone.0084729

**Published:** 2014-01-08

**Authors:** Huijuan Xu, Wen Qin, Hai Chen, Lin Jiang, Kuncheng Li, Chunshui Yu

**Affiliations:** 1 Department of Radiology and Tianjin Key Laboratory of Functional Imaging, Tianjin Medical University General Hospital, Tianjin, China; 2 Department of Neurology, Xuanwu Hospital of Capital Medical University, Beijing, China; 3 Department of Radiology, Xuanwu Hospital of Capital Medical University, Beijing, China; Katholieke Universiteit Leuven, Belgium

## Abstract

It remains uncertain if the contralesional primary sensorimotor cortex (CL_PSMC) contributes to motor recovery after stroke. Here we investigated longitudinal changes in the resting-state functional connectivity (rsFC) of the CL_PSMC and their association with motor recovery. Thirteen patients who had experienced subcortical stroke underwent a series of resting-state fMRI and clinical assessments over a period of 1 year at 5 time points, i.e., within the first week, at 2 weeks, 1 month, 3 months, and 1 year after stroke onset. Thirteen age- and gender-matched healthy subjects were recruited as controls. The CL_PSMC was defined as a region centered at the voxel that had greatest activation during hand motion task. The dynamic changes in the rsFCs of the CL_PSMC within the whole brain were evaluated and correlated with the Motricity Index (MI) scores. Compared with healthy controls, the rsFCs of the CL_PSMC with the bilateral PSMC were initially decreased, then gradually increased, and finally restored to the normal level 1 year later. Moreover, the dynamic change in the inter-hemispheric rsFC between the bilateral PSMC in these patients was positively correlated with the MI scores. However, the intra-hemispheric rsFC of the CL_PSMC was not correlated with the MI scores. This study shows dynamic changes in the rsFCs of the CL_PSMC after stroke and suggests that the increased inter-hemispheric rsFC between the bilateral PSMC may facilitate motor recovery in stroke patients. However, generalization of our findings is limited by the small sample size of our study and needs to be confirmed.

## Introduction

Motor disability is one of the most common deficits after stroke. Irrespective of the type and amount of therapy, nearly all stroke patients experience at least some predictable degree of functional recovery within the first six months after stroke [1], [2]. This spontaneous motor recovery may be associated with structural and functional reorganization of the motor network [3], [4]. In the first days to weeks after stroke, increased activity in both hemispheres has been reported during movements of the paretic hand [5]–[8]. Although this increased activation in the contralesional primary sensorimotor cortex (CL_PSMC) after stroke is highly consistent across experiments [9], its functional relevance is a matter of debate. The increased activation of the CL_PSMC has been considered to either facilitate [7], [10]–[14] or inhibit motor recovery [15]–[17]. Based on the theory of interhemispheric inhibition, the increased activation in the CL_PSMC is a result of reduced inhibition from the ipsilesional PSMC early after stroke [18]. Inhibiting the activity of the CL_PSMC by repetitive transcranial magnetic stimulation (rTMS) improves motor performance [19]–[22]. However, other groups have not observed the effect of motor improvement using the same method [23], [24]. On the contrary, a previous study reports deteriorated motor performance after inhibition of the CL_PSMC [25].

Resting-state functional connectivity (rsFC) reflects the temporal synchrony of functional MRI signals between remote regions and has been applied to stroke patients. Much attention has been paid to changes in the rsFCs of the ipsilesional PSMC after stroke [26]–[29]. Studies thus far have found that these rsFCs were initially decreased, then gradually increased during recovery, and were finally restored to near normal or above normal levels [26]–[29]. However, the dynamic changes in the rsFCs of the CL_PSMC after stroke remain unclear.

In the current study, we investigated dynamic changes in the rsFCs of the CL_PSMC and their correlations with motor recovery in subcortical stroke patients using a mixed-effects model to determine the functional relevance of the rsFCs of the CL_PSMC to the motor recovery.

## Subjects and Methods

### Subjects

Thirteen right-handed patients (all males; age 49.2±7.1 years, range 35–64 years) with subcortical ischemic stroke in the left (n = 8) or right (n = 5) motor pathways were enrolled from the inpatient service at the Xuanwu Hospital of Capital Medical University (Beijing, China). All patients were first-onset stroke patients who showed motor deficits in both the upper and lower extremities. None of them had a history of neurological or psychiatric disorders, nor did any experience subsequent symptomatic stroke. Conventional MR images did not show any abnormalities in the patients other than the infarct lesion. Thirteen age- and gender-matched right-handed healthy subjects (all males; age 49.6±6.8 years, range 35–64 years) were also recruited for comparison. The Ethics Committee of Xuanwu Hospital approved this study, and written informed consent was obtained from each patient or control subject.

**Figure 1:**
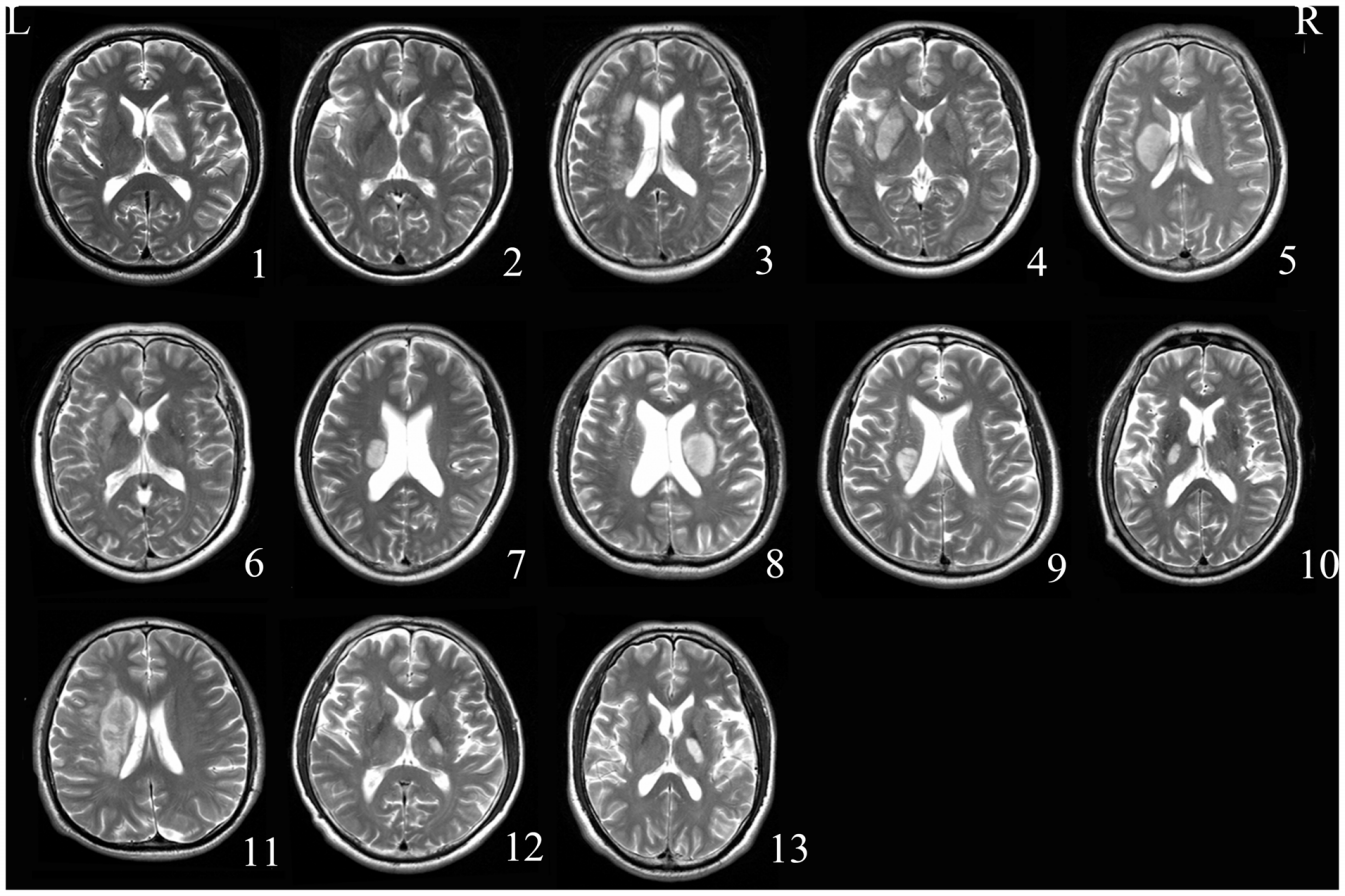
Lesion locations of stroke patients. The lesions are shown on axial slices of the T2-weighted images. Patient numbers correspond with those in Table 1. L, left and R, right.

The motor function of each patient was assessed using the Motricity Index (MI) [30] by Dr. Hai Chen who had 10-years experience in neurology. This scale measures motor abilities including hand-grasp, elbow flexion, shoulder abduction, ankle dorsiflexion, knee extension and hip flexion in the limbs on the affected side. The reliability and validity of this scale have been confirmed [31], [32]. The patients were scanned and clinically assessed at five consecutive time points, i.e. within the first week, at 2 weeks, 1 month, 3 months, and 1 year after stroke onset. The clinical characteristics and demographic data are summarized in Table 1 and the lesion of each stroke patient is shown in Figure 1.

**Table 1:** Demographic and clinical information of stroke patients.

PatientID	Age(years)	Lesion Side	Lesion Location	Lesion Volume(ml)	Time Points	Days after onset					Motricity Index				
						TP1	TP2	TP3	TP4	TP5	TP1	TP2	TP3	TP4	TP5
1	49	R	IC/BG	10.19	5	6	20	33	92	246	72	93.5	93.5	99	99
2	35	R	IC	2.78	4	5	14	67	118	–	75.5	91.5	91.5	95.5	–
3	42	L	IC/BG/CR	14.56	4	5	14	33	–	355	16.5	44	65	–	95
4	48	L	IC/BG/CR	14.72	5	2	13	36	89	302	0	7	9.5	41	47.5
5	53	L	IC/BG/CR	12.39	5	4	18	36	99	352	7	29	44	56.5	56.5
6	52	L	IC/BG	6.74	5	0	14	30	92	369	70.5	91.5	99	99	99
7	51	L	IC/BG/CR	3.72	4	7	16	–	61	414	7	18.5	–	44	58
8	64	R	IC/BG	10.80	4	6	15	30	–	315	42.5	50.5	58.5	–	83.5
9	50	L	IC	4.23	4	–	12	37	94	433	–	43	69	89.5	91.5
10	55	L	IC	2.51	4	5	13	32	60	–	18.5	26.5	45.5	51	–
11	41	L	IC/BG/CR	29.51	5	5	15	31	113	377	0	7	16.5	39	41.5
12	52	R	IC/Tha	1.67	4	–	15	24	93	352	–	23.5	33.5	41.5	41.5
13	48	R	IC	3.71	3	8	14	–	103	–	47	74.5	–	86.5	–
Mean±SD	49.2±7.1	–	–	9.04±7.74	–	4.8±2.7	14.8±2.2	35.4±16.7	92.2±38.4	351.5±161.1	32.4±30.0	46.2±31.9	56.9±35.0	67.5±34.7	71.3±35.5

IC, internal capsule; CR, corona radiate; BG, basal ganglia; Tha, thalamus, SD, standard deviation.

### MR Image Acquisition

All images were acquired using a Siemens Trio 3.0 Tesla MRI scanner (Siemens, Erlangen, Germany). Tight but comfortable foam padding was used to minimize head motion, and earplugs were used to reduce scanner noise. Functional MR images were collected using an echo-planar imaging (EPI) sequence with the following scan parameters: repetition time (TR)/echo time (TE) = 2000/30 ms; field of view (FOV) = 220 mm×220 mm; matrix = 64×64; flip angle (FA) = 90°; slice thickness = 3 mm; gap = 1 mm; 32 interleaved transversal slices; and 180 volumes. During the fMRI scans, all subjects were instructed to keep their eyes closed, stay as motionless as possible, think of nothing in particular, and not fall asleep. Structural images were obtained in the sagittal orientation by employing a magnetization prepared rapid gradient echo sequence over the whole brain: 176 slices, thickness/gap = 1.0/0 mm, matrix = 256×224, TR = 1600 ms, TE = 2.6 ms, FA = 9°, and FOV = 256 mm×224 mm. T2-weighted images were acquired using a turbo-spin-echo sequence: 20 axial slices, thickness/gap = 5.0/6.5 mm, matrix = 512×416, TR = 4140 ms, TE = 92 ms, and FOV = 187 mm×230 mm.

### Data Preprocessing

Before data preprocessing, we flipped the imaging data from right to left along the midsagittal line for the 5 patients who had lesions on the right hemisphere. For all patients, the left side corresponded to the ipsilesional hemisphere and the right side corresponded to the contralesional hemisphere. Preprocessing was performed using statistical parametric mapping (SPM8, http://www.fil.ion.ucl.ac.uk/spm). The first ten volumes of each subject were discarded to allow the signal to reach equilibrium and the participants to adapt to the scanning noise. The remaining 170 volumes were first corrected for the acquisition time delay between different slices and were then realigned to the first volume to correct for inter-scan movements. We controlled for head motion using a threshold of 2.5 mm translation in each cardinal direction and 2.5° rotation in each of the orthogonal x-, y- and z-axes. We also calculated the frame-wise displacement (FD), which indexes the volume-to-volume changes in head position that were obtained from derivatives of the rigid body realignment estimates that are used to realign BOLD data during fMRI preprocessing [33]. The average FD was considered as a nuisance covariate in the rsFC analyses. The realigned fMRI images were spatially normalized to Montreal Neurological Institute (MNI) space using the EPI template and were then re-sampled into a voxel size of 3×3×3 mm^3^. After normalization, the images were smoothed using a Gaussian kernel of 8×8×8 mm^3^ full-width at half maximum. Several sources of spurious variances from the estimated motion parameters, global average BOLD signals, and average BOLD signals in the ventricular and white matter regions were removed from the data through linear regression. Finally, temporal band-pass filtering (0.01–0.08 Hz) was performed on the time series of each voxel to reduce the effects of low-frequency drift and high-frequency noise [34]. Although global signal regression may partly reduce the effects of physiological noise and head motion, several studies have reported that global signal regression can influence the pattern of rsFC [35], [36]. To validate the robustness of our results, we also analyzed the fMRI data without global signal regression.

### Definition of Seed Region

**Figure 2:**
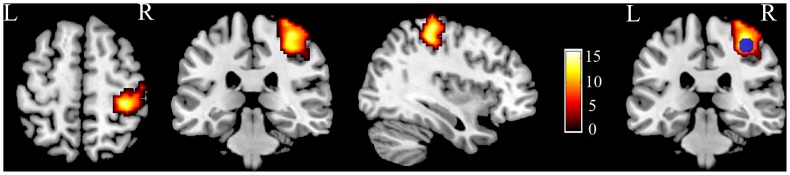
The BOLD response evoked by hand-grasping task. Red-yellow areas show activation of the right PSMC acquired by the left hand-grasping task of 11 normal controls, and the blue area indicates the seed region of the CL_PSMC. BOLD, blood oxygen level dependent; CL, contralesional hemisphere; L, left; PSMC, primary sensorimotor cortex; and R, right.

The seed region for rsFC analysis was defined as the CL_PSMC based on a previous work with a hand-grasping task using the left hand in 11 healthy subjects (4 females and 7 males; age 54.8±7.6 years).The motor task activated the right PSMC with a peak MNI coordinate of 36, −33, 54. The seed region of the CL_PSMC was defined as a sphere with a radius of 9 mm which centered at the peak MNI coordinates (Fig. 2) (Please see details in Method S1).

### Calculation of the rsFCs of the CL_PSMC

**Figure 3:**
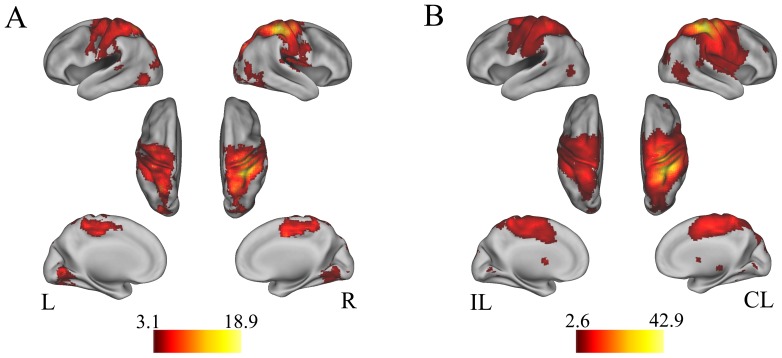
The resting-state functional connectivity patterns of the CL_PSMC. (A) Normal controls. (B) Stroke patients. CL, contralesional hemisphere; IL, ipsilesional hemisphere; L, left; PSMC, primary sensorimotor cortex; and R, right.

For each subject, Pearson correlation coefficients between the mean time course of the seed region and that of every voxel in the brain were computed and converted to z-values using Fisher’s r-to-z transformation to improve normality. Each individuals’ z-values were entered into a random-effect one-sample t-test in a voxel-wise manner to identify brain regions that showed positive correlations with the seed region (Fig. 3). Multiple comparisons were corrected using the False Discovery Rate (FDR, *P*<0.05) method with a cluster size of >30 voxels.

### Longitudinal Analysis

To investigate the dynamic change in the rsFCs between the CL_PSMC and other brain regions after stroke, a linear mixed model was employed to characterize monotonic changes in the rsFCs over time. The random intercept term accounts for the correlation due to repeated measurements within single patient [37]. This model might allow us to make the utmost use of all available data for each patient, even if some time points were missing. Each patient was assumed to possess a common slope (fixed effect) where only the intercepts were allowed to vary (random effect). The model was expressed as the following equation:
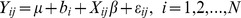
(1)where *Y_ij_* is each rsFC from the *j*th scan (up to five scans) of the *i*th patient (*i*≦13, *j*≦5); 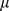 is the intercept term that is common to all subjects; 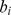 is a random intercept allowing a unique intercept for each patient; 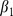 is the scalar of the fixed effect; *X_ij_* is the time interval (i.e., days after stroke); 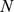 is the number of subjects; and 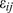 is the residual error of the model. The model parameters were estimated by the restricted maximum likelihood method and considered significant if the *P* values were <0.05.

To analyze the relationship between the rsFC changes and motor function after stroke, we also applied this mixed-effect model to investigate the correlations between changes in the rsFCs and motor recovery, in which *X_ij_* is the MI score from the *j*th time point of the *i*th patient post-stroke.

Using SPSS 13.0 for Windows (SPSS Inc, Chicago, IL, USA), we also compared the rsFCs of patients at different time points and healthy subjects. Here, a general linear model (GLM) was applied with individual variation for random factors and different time points for fixed factors (*P*<0.05). The GLM was also used to test the motor recovery (*P*<0.05) by treating the variation in the MI scores of each individual as random factor and different time points as fixed factor.

## Results

### Behavioral Data

**Figure 4:**
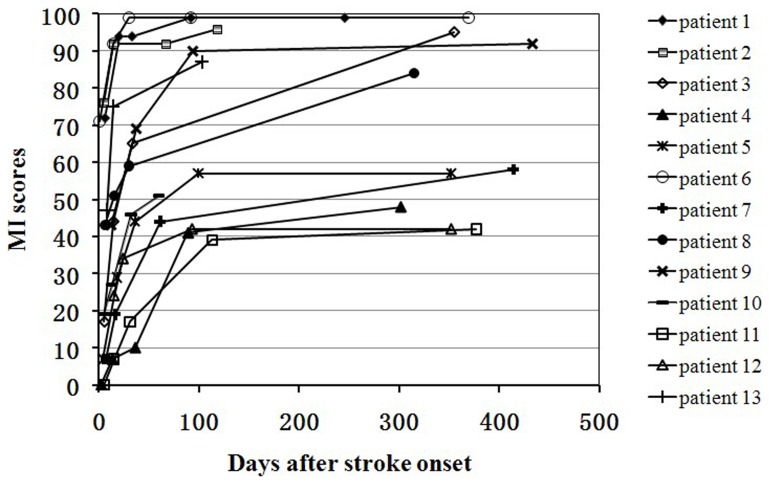
Post-stroke recovery curves of stroke patients. The x-axis denotes days after stroke onset; and the y-axis denotes MI scores (ranging from 0 to 100, 100 represents complete recovery). MI, motricity index.

Demographic and clinical characteristics of stroke patients are listed in Table 1. The time intervals between stroke onset and each session were as follows: session 1 (mean ± SD: 4±2 days post-stroke), session 2 (15±2 days), session 3 (35±11 days), session 4 (mean ± SD: 92±18 days), and session 5 (352±54 days). Five patients completed all 5 sessions, 7 patients finished 4 sessions, and 1 patient completed 3 sessions. The lesions are shown on T2-weighted images of the first session (Fig. 1). The average size of stroke lesions was 9.04±7.74 ml. The MI score of each time point is shown in Table 1. Based on the MI score, all patients demonstrated significant recovery (*P*<0.05), and the recovery curves of these stroke patients are shown in Fig. 4. The mean MI scores at 1 week, 2 weeks, 3 months and 1 year post stroke were 31.0±2.5, 46.2±2.3, 56.6±2.5, 68.3±2.5, and 75.7±2.7, respectively. The MI scores were significantly different between all time points (*P*<0.005), except between 3 months and 1 year post stroke (*P* = 0.056).

### Longitudinal Changes in the rsFCs of the CL_PSMC

**Figure 5:**
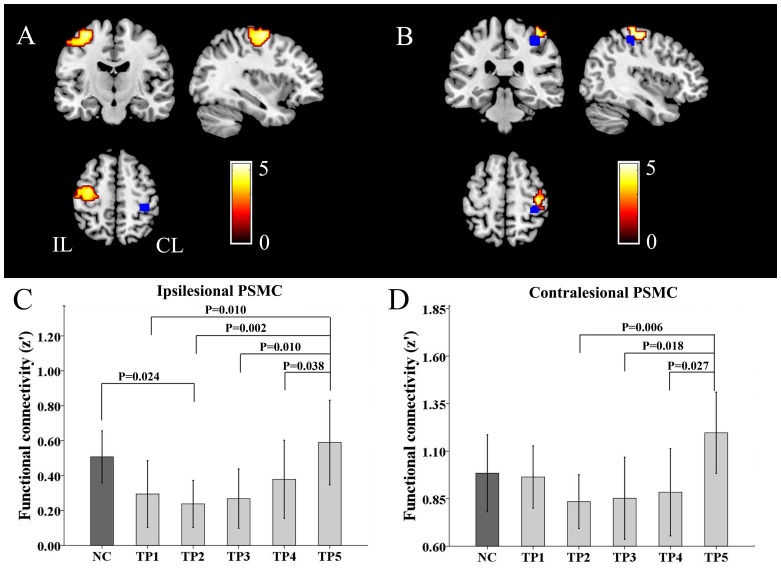
The changes in rsFC of the CL_PSMC. Brain regions show increased rsFCs with the CL_PSMC after stroke in the ipsilesional (A) and contralesional hemispheres (B). (C) and (D) show group comparisons of inter-hemispheric and intra-hemispheric rsFC of healthy subjects and patients at 5 time points. Bars represent the means, error bars represent the SDs, and stars represent the significant differences between groups. NC, normal control; TP, time points; CL, contralesional hemisphere; IL, ipsilesional hemisphere; PSMC, primary sensorimotor cortex; and rsFC, resting-state functional connectivity.

In the voxel-based rsFC analysis, the bilateral PSMC exhibited linearly increased rsFCs with the CL_PSMC after stroke (Fig. 5 and Table 2), but no brain region showed significantly decreased rsFC with the CL_PSMC after stroke (Table 2). We further compared the strengths of the rsFCs across healthy controls and patients at different time points (Fig. 5). Compared with healthy controls, stroke patients showed a decreased interhemispheric rsFC between the bilateral PSMC immediately after stroke onset that reached the lowest level at two weeks. This decreased rsFC restored to a near normal level at 3 months post-stroke and then increased to the normal level 1 year after stroke (Fig. 5C). When comparing intrahemispheric rsFC of the CL_PSMC of stroke patient to those of healthy control, the rsFC did not show significant decrease within one week and slightly decreased at two weeks. Subsequently, this rsFC was gradually increased to the near or above normal level (Fig. 5D).

**Table 2:** Brain regions show significant linear changes in the rsFC within the CL_PSMC after stroke.

Brain regions	Peak*t-*value	Cluster Size(voxels)	MNI Coordinates		
			x	y	z
Ipsilesional PSMC	5.48	205	−33	−22	63
Contralesional PSMC	4.27	48	39	−21	60

CL, contralesional hemisphere; MNI, Montreal Neurological Institute; PSMC, primary sensorimotor cortex; and rsFC, resting-state functional connectivity.

Note: Both brain regions show increased rsFC with the CL_PSMC after stroke, whereas no brain regions show decreased rsFC with the CL_PSMC after stroke.

### The Associations of rsFC Longitudinal Changes with Clinical Scores

**Figure 6:**
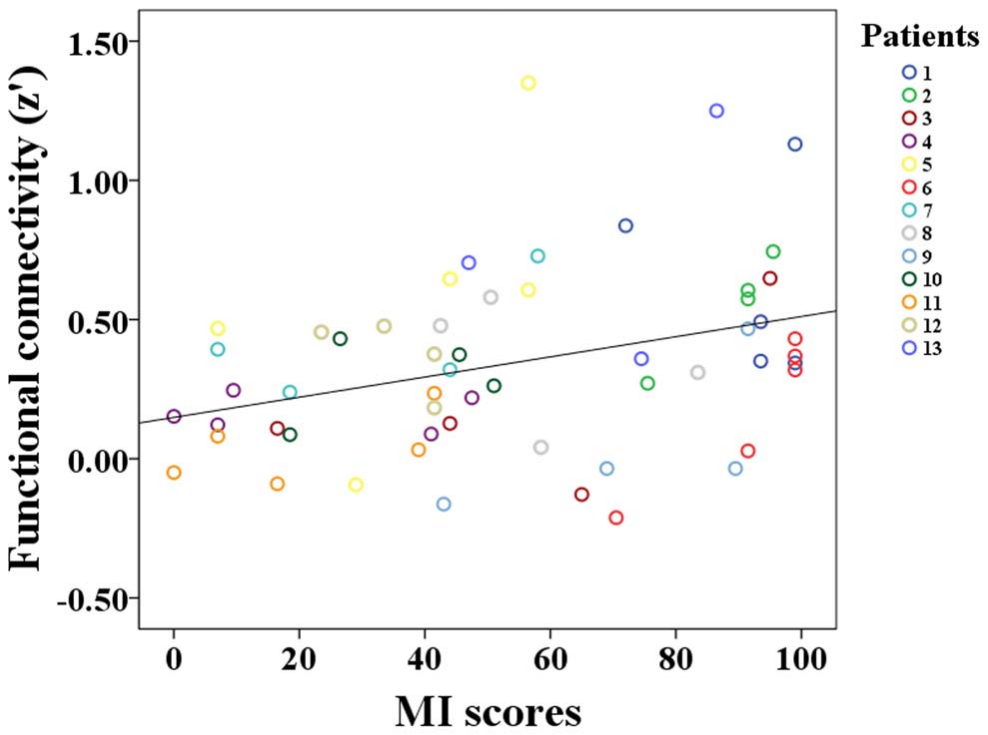
Correlation between the inter-hemispheric rsFC of the PSMC and MI scores after stroke. The x-axis represents MI scores at each time point after stroke, and the y-axis denotes the rsFC of the bilateral PSMC. CL, contralesional hemisphere; IL, ipsilesional hemisphere; MI, motricity index; PSMC, primary sensorimotor cortex; and rsFC, resting-state functional connectivity.

A mixed-effects model was applied to further explore the relationship between the rsFC and motor performance after stroke. A positive correlation with MI scores was found only in the rsFC between the bilateral PSMC (*t = *2.28, P = 0.028) during stroke recovery (Fig. 6), and no significant correlations (*t = *1.24, P = 0.223) were evident between the MI scores and intrahemispheric rsFC of the CL_PSMC.

### The Effect of Global Signal Regression on the Results

The above-mentioned analyses were repeated on the fMRI data with the same preprocessing steps but without global signal regression. The results were described in Result S1 and the difference between both analyses was judged visually. The results derived from the fMRI data without global signal regression were similar to those after global signal regression (Figures S1–S6).

## Discussion

To our knowledge, this is the first longitudinal study aimed to elucidate the functional relevance of the rsFCs of the CL_PSMC with motor recovery after subcortical stroke. We found that only a dynamic change in the inter-hemispheric rsFC between the bilateral PSMC was associated with motor recovery in stroke patients. This finding is in line with a previous study that reported increased rsFC of the motor network in stroke patients following an electroencephalography-based brain computer interface training program with motor imagery [38]. It should be noted that only patients with subcortical stroke were included in this study to improve the homogeneity of the patient population because cortical+subcortical and subcortical stroke patients have been shown to use completely different neural mechanisms for motor recovery [39], [40].

### Decreased rsFCs of the CL_PSMC at the Acute Stage of Stroke

We found decreased rsFCs between the bilateral PSMC at acute stage of stroke, which is consistent with prior findings from both rats and humans. In stroke rats with lesions that comprised subcortical and cortical tissue [36], [37], the rsFC between the bilateral PSMC significantly decreased during the first days after stroke [41], which is paralleled by sensorimotor function deficits. Moreover, the reduced inter-hemispheric rsFC in these stroke rats was also accompanied by a decrease in transcallosal manganese transfer between these regions [42]. The latter finding reflects anatomical disconnection, which may underlie the impaired inter-hemispheric rsFC. Consistent with these results from animal experiments, an early decrease in the inter-hemispheric rsFC of the motor system of humans has been reported not only in subcortical stroke patients [26], [28], [29], [43] but also in stroke patients with cortical+subcortical lesions [26], [27]. The rsFC impairment between inter-hemispheric homogeneous regions is not confined to the motor system. In stroke patients with spatial neglect [26], [44] and aphasia [45], reduced inter-hemispheric rsFC was also found between areas related to attention or language.

At acute stage of stroke, we found reduced inter-hemispheric rsFC, which has been correlated with impairment of the corticospinal tract (CST) in subcortical stroke patients [43], [46]. At this stage, the bilateral PSMC remain relatively intact because subcortical lesions require months to affect the cortical regions via the retrograde degeneration mechanism [47]. Thus direct cortical impairments cannot explain for the reduced inter-hemispheric rsFC at this stage. The most possible explanation for this finding is inter-hemispheric diaschisis, which may transiently influence electrical activity, blood flow, metabolism, and possibly the rsFC [48], [49].

### Longitudinal Changes in the Inter-hemispheric rsFC of the PSMC after Stroke

After the initial reduction in the rsFC between the bilateral PSMC, the rsFC gradually increased during the recovery progress following stroke. More importantly, the linearly increased inter-hemispheric rsFC was correlated with motor recovery in these patients. In stroke rats with lesions that comprised subcortical and cortical tissue [41], the initially decreased rsFC between the bilateral PSMC was gradually restores and the temporal pattern of changes in the inter-hemispheric rsFC was significantly correlated with the evolution of sensorimotor function scores [41]. Subsequently, these authors found that the degree of functional recovery after stroke was associated with the extent of reinstatement of the inter-hemispheric rsFC [46]. Similar dynamic evolution and functional relevance of the rsFC between the bilateral PSMC have been observed in stroke patients with subcortical lesions [28] and lesions in cortical and subcortical tissue [26]–[29]. Although the neural mechanisms for the reinstatement of the inter-hemispheric rsFC remain unclear, it may be related to the disappearance of the transient transhemispheric diaschisis [49] and axons sprouting to establish new connections and projections [50], [51].

### Longitudinal Changes in the Intra-hemispheric rsFC of the CL_PSMC after Stroke

We also found a trend of an initial reduction and subsequent increase in the intra-hemispheric rsFC of the CL_PSMC following subcortical stroke. The increased intra-hemispheric rsFC within the CL_PSMC has also been previously reported in stroke rats with both subcortical and cortical lesions [41], [42]. The increased intra-hemispheric rsFC within the CL_PSMC at one year post-stroke may be associated with the remodeling of neuronal elements, i.e., axonal sprouting, synaptogenesis, and dendritic growth, which has been detected in various animal stroke models [3], [52]. We demonstrated that the intra-hemispheric rsFC of the CL_PSMC did not correlate with the restoration of motor function and was not significantly different from those of the healthy controls at any time point. These findings are consistent with previous reports on the non-significant contribution of intrahemispheric rsFC to motor recovery in stroke patients [26]. Of course, we cannot exclude the possibility that our monotonic linear model is not the best one for modeling association between changes in intra-hemispheric rsFC of the CL_PSMC and motor recovery. Further studies should be performed to determine the best statistical model for modeling longitudinal changes of the MRI and behavioral measures after stroke.

### The Functional Relevance of the CL_PSMC to Motor Recovery after Stroke

It seems contradictory that the motion-elicited-activation of the affected hand in the CL_PSMC is initially increased and then gradually reduced to the normal level [7], [53], whereas the rsFCs of the CL_PSMC are initially reduced and then gradually increased to the normal level. The nearly inversed patterns in activation changes and rsFC of the CL_PSMC make it difficult to infer the functional relevance of the CL_PSMC in motor recovery after stroke. Actually, the debate on the functional relevance of the CL_PSMC in motor recovery also comes from other contradictory findings obtained by fMRI and TMS studies [18]–[25]. Although the activation of the CL_PSMC was reported to play an important role in subcortical stroke patients performing complex motor tasks [8], [17], [25], [54], this increased activation is more frequently reported in stroke patients with poor recovery [17], [49] and negatively correlates with motor recovery [48], [49]. Moreover, effective connectivity and TMS studies on interhemispheric inhibition suggested that the CL_PSMC exerted increased inhibitory influences on ipsilesional PSMC in subcortical stroke patients, thus preventing patients from motor recovery [55]–[57]. The complex changes in the functional characteristics of the CL_PSMC may depend on multiple factors, such as the extent of damage in the motor cortex, time intervals after stroke, and task complexity. Severe damage of the motor cortex (such as cortical stroke) may predict the increased activation of the CL_PSMC during the movement of the affected hand, suggesting a compensatory role in motor recovery. In contrast, in stroke patients with a relatively intact motor cortex (such as subcortical stroke), the initially increased activation of the CL_PSMC may compensate for the insult; however, the persistent high activity in the CL_PSMC, especially at the recovery stage, may exert an increased inhibitory influence on ipsilesional PSMC, thereby preventing patients from motor recovery. In stroke patients, the increased activation of the CL_PSMC during complex motor tasks suggests that more neural resources should be recruited in this situation.

The inter-hemispheric rsFC of the PSMC may play a crucial role in the bimanual coordination of motor execution [58], [59]. The initial reduction of this rsFC may indicate functional dysfunction that is caused by transient transhemispheric diaschisis [49]. The subsequent reinstatement of the inter-hemispheric rsFC may represent functional recovery, which is associated with the disappearance of the transient transhemispheric diaschisis and the formation of new connections [50], [51].

### Limitations

One limitation of this study is the relatively small sample size and percentage of dropouts from the long-term follow-up; thus, further validation of our findings is necessary. The small sample size also prevents us from evaluating the influence of the severity of initial motor impairment on the longitudinal brain reorganization after stroke. Although only stroke patients with subcortical lesions were included, the relatively large difference in lesion location and volume might have impacted on current study results. However, correlation analysis did not show any significant correlations between lesion volume and the rsFC of the CL_PSMC or MI scores at each time point. Longitudinal follow-up of a larger study sample with lesion subgroups is required to verify current study results. The MI is a quick and easy to administer assessment scale, though it only provides a crude scoring of a patient’s motor function. Moreover, we did not distinguish upper limb from lower limb recovery, which are known to exhibit a different recovery pattern [60]. Since global signal regression may influence the rsFC analysis [35], [36], we tested the effects of global signal regression on our results and did not find any significant differences between the results with and without global signal regression, suggesting the robustness of our findings.

### Conclusions

Using a longitudinal design, we investigated the dynamic changes in the rsFCs of the CL_PSMC after subcortical stroke and found that both the inter-hemispheric and intra-hemispheric rsFCs of the CL_PSMC experienced significant changes. We also found that only the inter-hemispheric rsFC of the PSMC correlated with motor recovery. Our findings suggest that monitoring the rsFC between the bilateral PSMC may be a potential tool for evaluating and predicting the motor recovery of subcortical stroke patients.

## Supporting Information

Figure S1 **The rsFC pattern of the PSMC in normal control without global signal regression (** ***q*** **<0.05; FDR corrected).** CL, contralesional hemisphere; FDR, false discovery rate; IL, ipsilesional hemisphere; PSMC, primary sensorimotor cortex; and rsFC, resting-state functional connectivity.
(TIF)

Figure S2 **The rsFC pattern of the CL_PSMC in subcortical stroke patients without global mean regression (** ***q*** **<0.05; FDR corrected).** CL, contralesional hemisphere; FDR, false discovery rate; IL, ipsilesional hemisphere; PSMC, primary sensorimotor cortex; and rsFC, resting-state functional connectivity.
(TIF)

Figure S3 **The rsFC pattern of the PSMC in normal control with global signal regression (** ***q*** **<0.05; FDR corrected).** CL, contralesional hemisphere; FDR, false discovery rate; IL, ipsilesional hemisphere; PSMC, primary sensorimotor cortex; and rsFC, resting-state functional connectivity.
(TIF)

Figure S4 **The rsFC pattern of the CL_PSMC in subcortical stroke patients with global mean regression (** ***q*** **<0.05; FDR corrected).** CL, contralesional hemisphere; FDR, false discovery rate; IL, ipsilesional hemisphere; PSMC, primary sensorimotor cortex; and rsFC, resting-state functional connectivity.
(TIF)

Figure S5 **The linear changes of the rsFCs of the CL_PSMC after subcortical stroke.** (A) The result without global signal regression of the resting-state fMRI data (*q* <0.05; FDR corrected); (B) Overlapping of the results with (yellow) and without (red) global signal regression. CL, contralesional hemisphere; FDR, false discovery rate; IL, ipsilesional hemisphere; PSMC, primary sensorimotor cortex; and rsFC, resting-state functional connectivity.
(TIF)

Figure S6 **ROI-based comparisons of the significant rsFC across time points using fMRI data without global mean regression while with FD regressed.** FD, frame-wise displacement; NC, normal control; PSMC, primary sensorimotor cortex; rsFC, resting-state functional connectivity; and TP, time points.
(TIF)

Method S1 Definition of seed region of the contralesional primary sensorimotor cortex.
(DOC)

Result S1 Analyzing Results without global signal regression.
(DOC)
