# Supplementary figures and images for: Contribution of the Resting-State Functional Connectivity of the Contralesional Primary Sensorimotor Cortex to Motor Recovery after Subcortical Stroke
Source: PLoS One. 2014 Jan 8;9(1):e84729. doi: 10.1371/journal.pone.0084729 (PMC3885617; doi:10.1371/journal.pone.0084729)

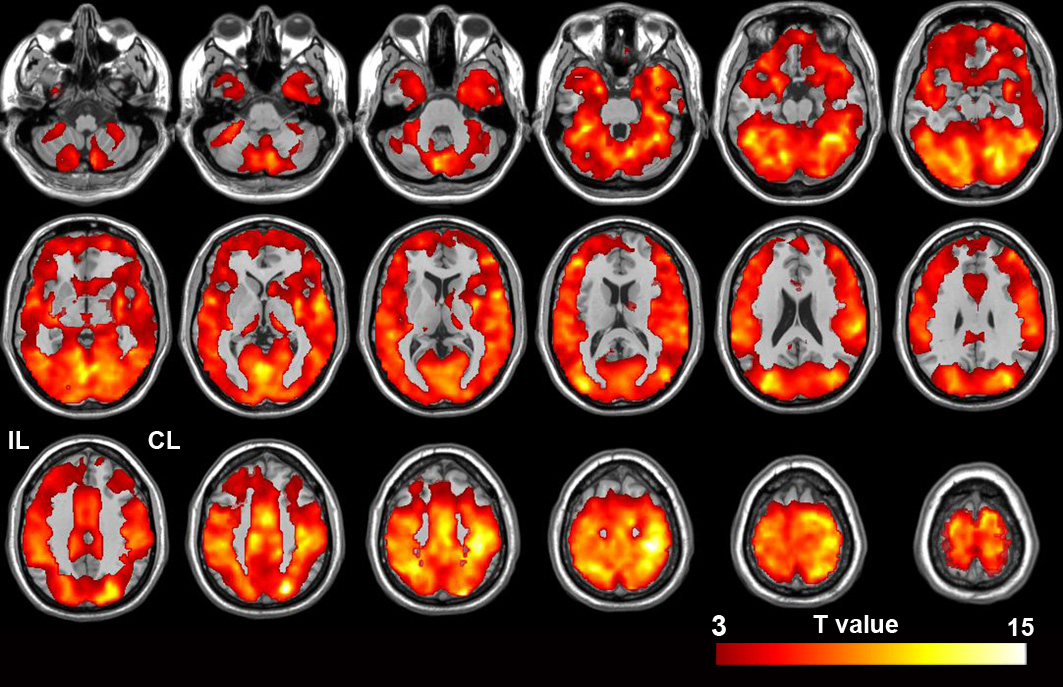

Supplement: Figure S1 — The rsFC pattern of the PSMC in normal control without global signal regression ( q <0.05; FDR corrected). CL, contralesional hemisphere; FDR, false discovery rate; IL, ipsilesional hemisphere; PSMC, primary sensorimotor cortex; and rsFC, resting-state functional connectivity. (TIF) [file pone.0084729.s001.tif]

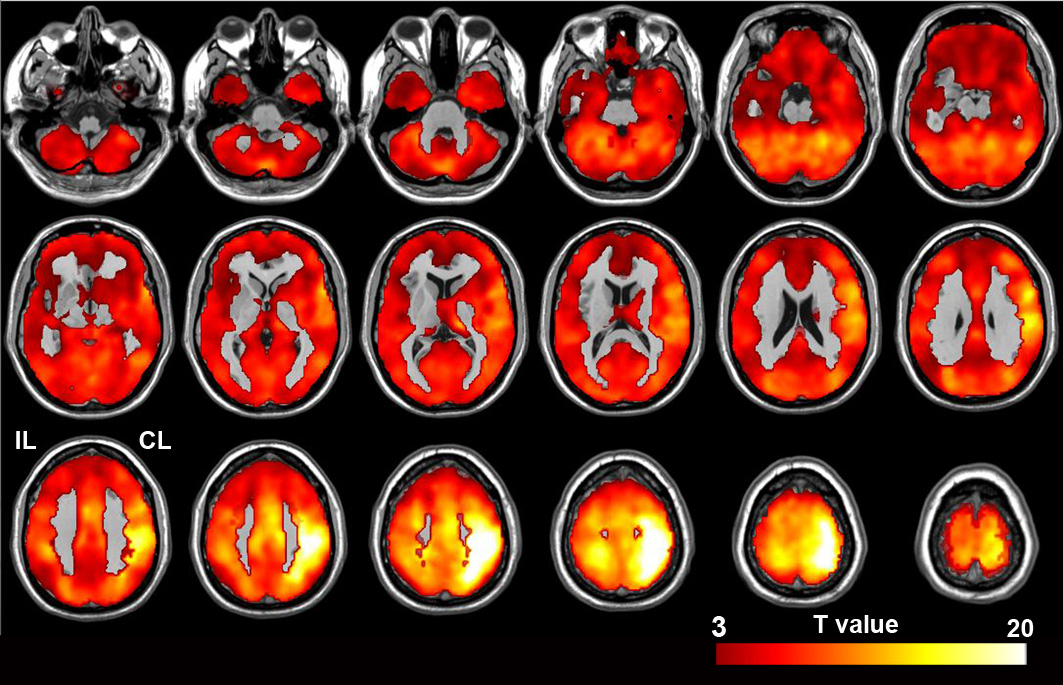

Supplement: Figure S2 — The rsFC pattern of the CL_PSMC in subcortical stroke patients without global mean regression ( q <0.05; FDR corrected). CL, contralesional hemisphere; FDR, false discovery rate; IL, ipsilesional hemisphere; PSMC, primary sensorimotor cortex; and rsFC, resting-state functional connectivity. (TIF) [file pone.0084729.s002.tif]

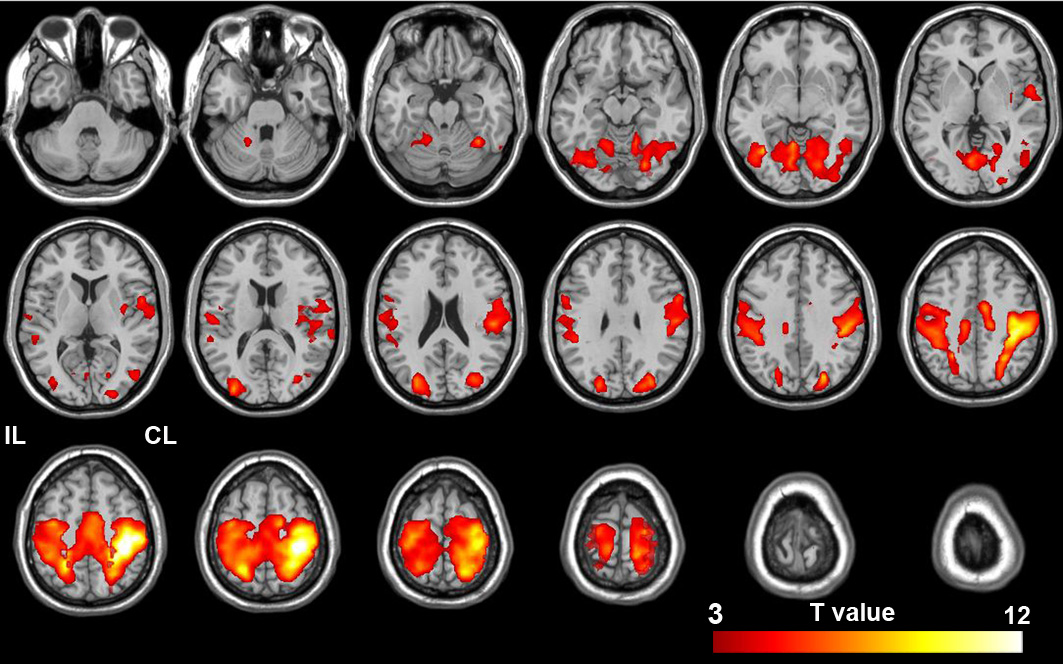

Supplement: Figure S3 — The rsFC pattern of the PSMC in normal control with global signal regression ( q <0.05; FDR corrected). CL, contralesional hemisphere; FDR, false discovery rate; IL, ipsilesional hemisphere; PSMC, primary sensorimotor cortex; and rsFC, resting-state functional connectivity. (TIF) [file pone.0084729.s003.tif]

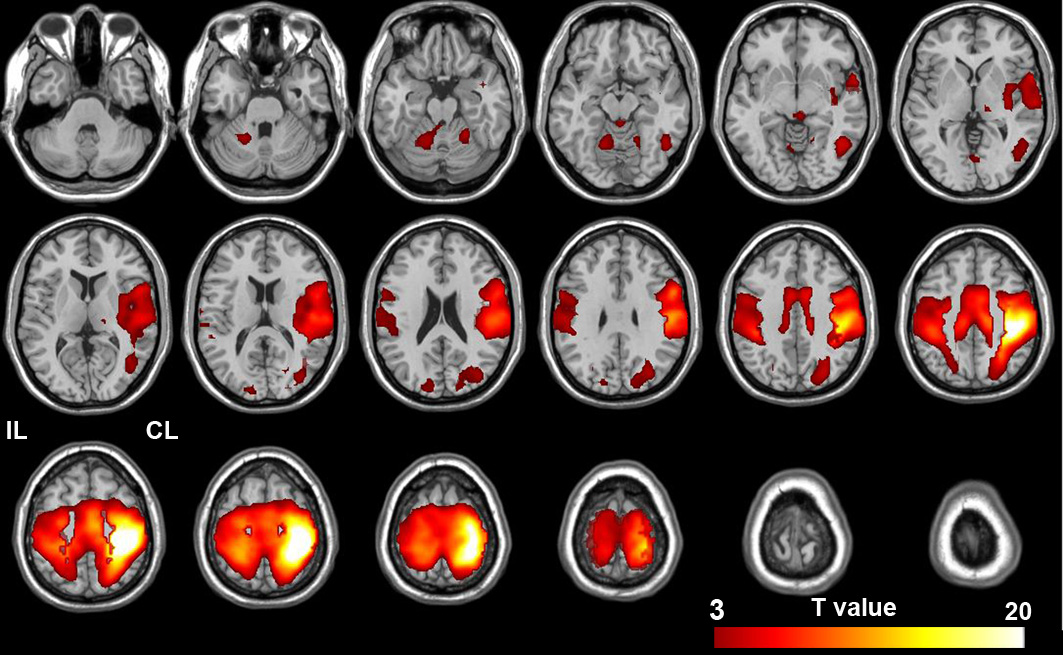

Supplement: Figure S4 — The rsFC pattern of the CL_PSMC in subcortical stroke patients with global mean regression ( q <0.05; FDR corrected). CL, contralesional hemisphere; FDR, false discovery rate; IL, ipsilesional hemisphere; PSMC, primary sensorimotor cortex; and rsFC, resting-state functional connectivity. (TIF) [file pone.0084729.s004.tif]

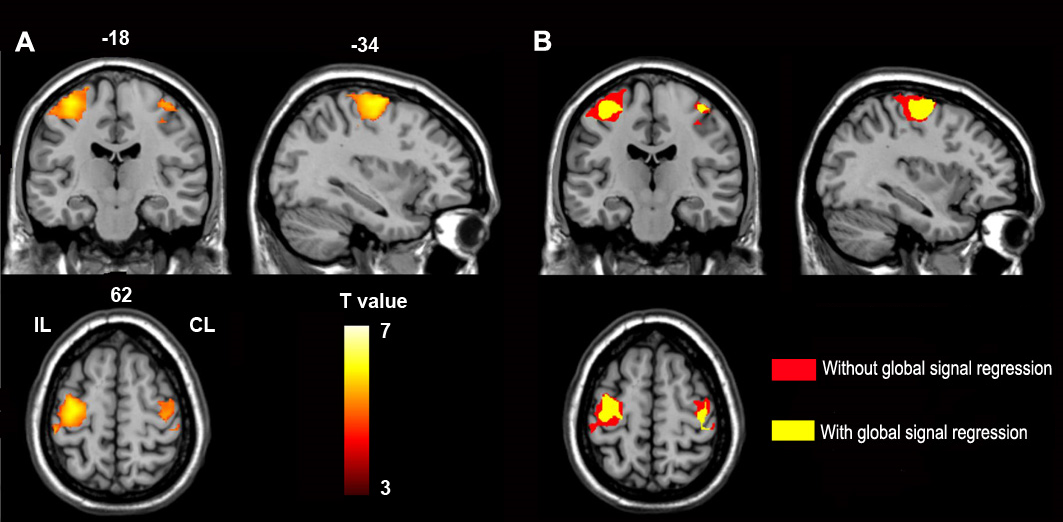

Supplement: Figure S5 — The linear changes of the rsFCs of the CL_PSMC after subcortical stroke. (A) The result without global signal regression of the resting-state fMRI data (q <0.05; FDR corrected); (B) Overlapping of the results with (yellow) and without (red) global signal regression. CL, contralesional hemisphere; FDR, false discovery rate; IL, ipsilesional hemisphere; PSMC, primary sensorimotor cortex; and rsFC, resting-state functional connectivity. (TIF) [file pone.0084729.s005.tif]

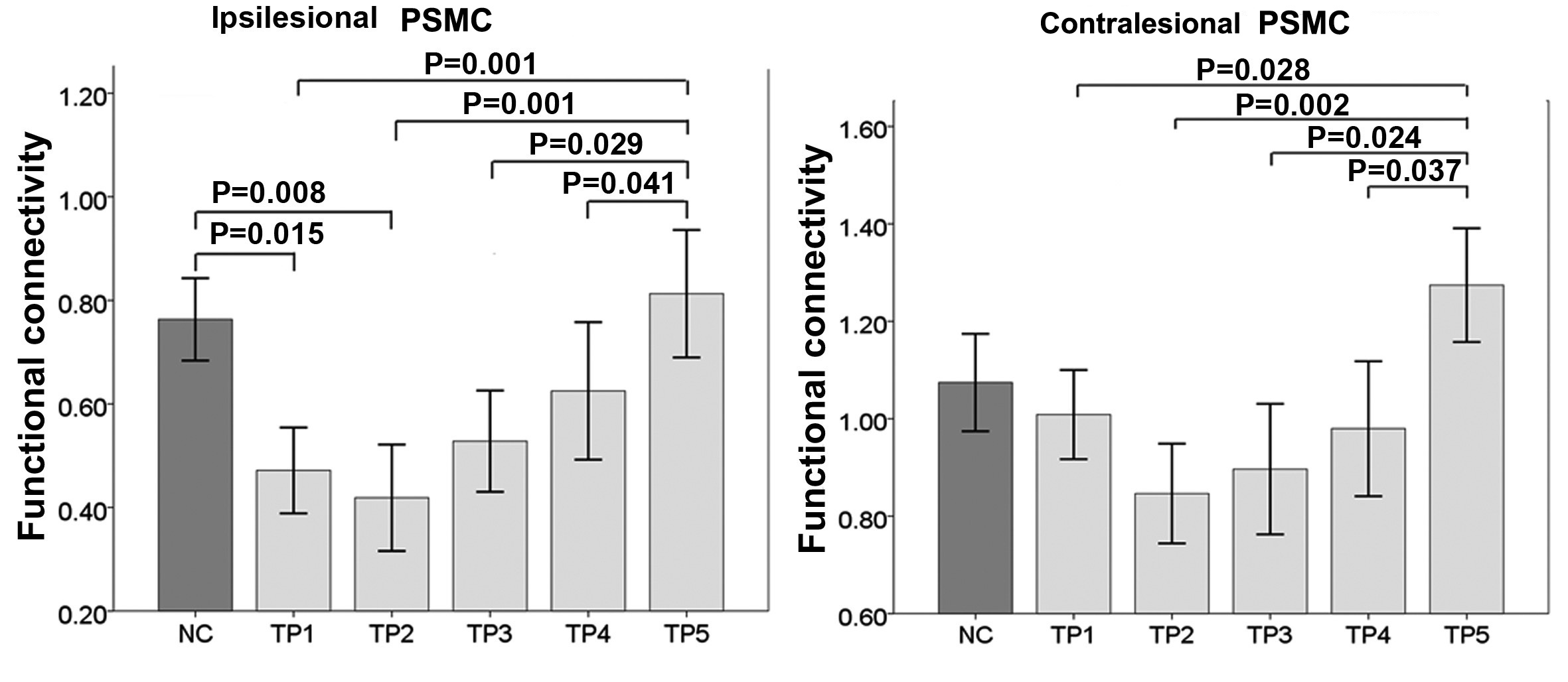

Supplement: Figure S6 — ROI-based comparisons of the significant rsFC across time points using fMRI data without global mean regression while with FD regressed. FD, frame-wise displacement; NC, normal control; PSMC, primary sensorimotor cortex; rsFC, resting-state functional connectivity; and TP, time points. (TIF) [file pone.0084729.s006.tif]
