# Supplementary material for: Contribution of the Resting-State Functional Connectivity of the Contralesional Primary Sensorimotor Cortex to Motor Recovery after Subcortical Stroke
Source: PLoS One. 2014 Jan 8;9(1):e84729. doi: 10.1371/journal.pone.0084729 (PMC3885617; doi:10.1371/journal.pone.0084729)
Supplement: Method S1 — Definition of seed region of the contralesional primary sensorimotor cortex. (DOC) [file pone.0084729.s007.doc]

**Definition of seed region of the contralesional primary sensorimotor cortex (CL_PSMC)**

The seed region of the CL_PSMC was defined as the most significant activation region during a hand motion task using the left hand in 11 healthy subjects (4 females; age: 54.8 ± 7.6 years). The 11 healthy volunteers were recruited from the community. They were undergone a detailed interview as well as neurological examinations. None of them reported a history of any neurological, psychiatric or cardiac disease or medication of centrally active drugs. They were right-handed according to the Edinburgh handedness inventory. Informed consent was obtained from each subject in accordance with the Ethical Committee of Tianjin Medical University General Hospital.

These subjects performed a unilateral voluntary hand-grasping task with a frequency of 2.4 Hz. A cycle of a 20 s resting block (baseline), a 20 s unilateral hand grasping block was repeated four times using their left hands. Instructions for start and end of each block were given by visually cue on a video screen. Before MR scanning, these subjects were trained to perform the hand-grasping task at the ﬁxed rate (2.4 Hz) without movement of the wrist.

Functional MR images were acquired using a Signa HDx 3.0 Tesla MR scanner (Trio, 8 high-frequency head coil; General Electric, Milwaukee, WI, USA) with 38 oblique transverse slices (3 mm thickness, 1 mm gap) covering the entire head using a gradient echo-planar imaging (EPI) sequence [repetition time (TR) /echo time (TE)=2000/30 ms; matrix size=64× 64; flip angle=90°; field of view (FOV) = 240 mm × 240 mm, 80 volumes]. Tight but comfortable foam padding was used to minimize head motion, and earplugs were used to reduce scanner noise.

The task fMRI data were analyzed using SPM8 (http://www.fil.ion.ucl.ac.uk/spm/) running on a mathematical platform (MATLAB 7.8.0; The Mathworks, Inc, Natick, Massachusetts). The fMRI data of each subject were corrected for acquisition time delay between slices and then spatially realigned. The motion-corrected functional volumes were spatially normalized to Montreal Neurological Institute (MNI) space using EPI template and then re-sampled into a voxel size of 3× 3 × 3 mm^3^. After normalization, images were smoothed using a Gaussian kernel of 8 × 8 × 8 mm^3^ full-width at half-maximum. For within-subject level (first level) analysis, The smoothed BOLD time series were convolved with the canonical hemodynamic response function and a high-pass ﬁlter with 128 s was applied to eliminate signal drifts.Then task-related activity were identified using the general linear model. Finally, the task-related activations of all subjects were entered into a second-level random-effects analysis. Group activation regions for left hand motion were determined using a false discovery rate (FDR) correction (*p* < 0.005) and a cluster size of > 30 voxels.

The motor task activated the right PSMC with a peak MNI coordinate of 36, -33, 54. The seed region of the CL_PSMC was defined as a sphere with a radius of 9 mm which centered at the peak MNI coordinates.
