# Supplementary material for: Contribution of the Resting-State Functional Connectivity of the Contralesional Primary Sensorimotor Cortex to Motor Recovery after Subcortical Stroke
Source: PLoS One. 2014 Jan 8;9(1):e84729. doi: 10.1371/journal.pone.0084729 (PMC3885617; doi:10.1371/journal.pone.0084729)
Supplement: Result S1 — Analyzing Results without global signal regression. (DOC) [file pone.0084729.s008.doc]

**Analyzing Results without global signal regression**

Global signal is the average signal across all voxels in the brain and is assumed to reflect a combination of resting-state fluctuations, physiological noise (respiratory and cardiac noise), and other noise signals with non-neural origin, such as head motion. In the present study, global signal regression was used to reduce the effects of physiological noise and head motion. However, several studies have shown that global signal regression can dramatically influence the pattern of resting-state functional connectivity (rsFC) , which may affect the group analysis in this study. In order to clarify this methodological issue, we repeated the preprocessing steps with the same parameters as the original one but without global signal regression.

First, we investigated the rsFC pattern of the contralesional primary sensorimotor cortex (CL_PSMC) in stroke patients and normal controls without global signal regression. Using the same statistical threshold as the original one, we found that the rsFC map of the CL_PSMC derived from fMRI data without global signal regression was extended much beyond the range of the sensorimotor network (SMN) and included almost all of the cortical areas of the brain (Figure S1 and S2). However, using fMRI data after global signal regression, we found that the rsFC map of the CL_PSMC was mainly restricted to the SMN (Figure S3 and S4).

Second, we test if the result of dynamic change of the rsFC of the CL_PSMC after stroke is influenced by global signal regression using the same linear mixed model. We found that the ipsilesional and contralesional PSMC also showed significantly linearly increased rsFCs with the CL_PSMC after stroke (Figure S5A). These results were highly overlapped with the results with global signal regression (Figure S5B).

Third, we extract the brain regions with significant linear changes after stroke derived from fMRI data without global mean regression, and put them into group comparisons with normal controls and between different time points. As shown in Figure S6, the result derived from fMRI data without global mean regression was highly consistent with that with global mean regression. Namely, compared with healthy controls, stroke patients showed decreased interhemispheric rsFC between the bilateral PSMC immediately after stroke onset and reached the lowest at two weeks. This decreased rsFC restored to the near normal level at 3 months post-stroke and then gradually increased to the normal level at 1 year after stroke. While the rsFC did not show significant decrease within 1 week and slightly decreased at two weeks for the intrahemispheric rsFC of the seed relative to the healthy controls. After that, this rsFC was gradually increased to the near or above normal level.

**Supplementary References：**

Murphy, K., Birn, R.M., Handwerker, D.A., Jones, T.B., Bandettini, P.A., 2009. The impact of global signal regression on resting state correlations: are anti-correlated networks introduced? Neuroimage 44, 893-905.

Saad, Z.S., Gotts, S.J., Murphy, K., Chen, G., Jo, H.J., Martin, A., Cox, R.W., 2012. Trouble at rest: how correlation patterns and group differences become distorted after global signal regression. Brain Connect 2, 25-32.
